# Clinical analysis of nine cases of uterine intravenous leiomyomatosis and review of the literature

**DOI:** 10.1097/MD.0000000000048251

**Published:** 2026-04-24

**Authors:** Junling Liu, Qiucheng Jia, Xiuwen Zhang, Huimin Tang, Yuqing Cheng, Hong Zheng, Yafeng Zheng, Jiming Chen, Na Zhang

**Affiliations:** a Department of Gynecology, The Second People’s Hospital of Changzhou, The Third Affiliated Hospital of Nanjing Medical University, Changzhou, Jiangsu, China; b Department of Pathology, The Second People’s Hospital of Changzhou, T he Third Affiliated Hospital of Nanjing Medical University, Changzhou, Jiangsu, China; c Department of Gynecology, People’s Hospital of Ningxia Hui Autonomous Region, Ningxia Medical University, Yinchuan, Ningxia, China.

**Keywords:** diagnosis, hysterectomy, intravenous leiomyomatosis, myomectomy

## Abstract

Intravenous leiomyomatosis (IVL) is a rare and special type variant of leiomyoma characterized by intravascular proliferation of benign smooth muscle cells extending beyond the uterus into the heart and even to the pulmonary arteries through a variety of ways. Owing to its rare occurrence and lacking of obvious symptoms, early diagnosis is very difficult. Complete excision of tumors is vital for a favorable prognosis. There is no consensus about the operative drug-assisted treatment to reduce the risk of recurrence. Pathogenesis of IVL is not clear, and chromosomal and genetic changes may accelerated the development of IVL. Herein, we report 9 patients with early stage IVL. Nine patients were admitted to the hospital with different symptoms for surgical treatment, and postoperative pathology was suggestive of intravascular smooth muscle tumor. Close follow-up of the patient post-operatively revealed that hysterectomy and bilateral adnexectomy could be chosen when the mass was limited to the uterus or pelvic cavity, and for patients with fertility requirements, myomectomy might also be feasible. About hormonal or the other adjuvant therapies after operation, there are still controversies, and further studies are needed to verify these findings. We retrospectively analyzed the clinical data of the patients and explored their clinical features, treatment and prognosis, aiming to improve the understanding of this disease.

## 1. Introduction

Intravenous leiomyomatosis (IVL) is a special type of uterine leiomyoma with fewer than 300 cases described in literature.^[1]^ The first case was reported in 1896.^[2]^ However, even if such tumors are histologically confirmed to be benign, but biological behavior often presents clinically aggressive. The tumor can not only develop in the intrauterine veins, but also spread into the systemic veins and even right atrium, pulmonary arteries. Although a rare neoplasm, IVL can lead to various degrees of vascular occlusion and bring about death. When the tumor extend into cardiac chambers, it may result in heart failure and atrioventricular incarceration,^[2]^ and thus cause sudden death. Due to the variable clinical presentation, IVL is easily misdiagnosed and missed diagnosed. Multiple imaging modalities, such as enhanced computed tomography (CT) imaging, magnetic resonance imaging (MRI), B-ultrasonography and so on, are vital to preoperative diagnosis of IVL. Because of its high rate of recurrence, many reported cases perform complete resection, sometimes appropriate drug-assisted treatment also may be used after surgery. But there is no apparent consensus for the optimal approach to surgical resection at present, and there is still controversy about the effectiveness of postoperative drug-assisted treatment. In recent years, many studies on pathogenic genes of IVL are increasing, and some of these genes are related to the recurrence of this disease. The achievements is expected to provide an effective targeted therapy for IVL and prolong the survival period of patients.

In this study, we reported 9 patients with IVL belonging to early stage, who have not obvious clinical symptoms. Besides, we review the literature to increase the understanding of this disease and provide an argument for management of IVL. The present study was approved by the ethics committees of the affiliated Changzhou No. 2 People’s Hospital of Nanjing Medical University. All 9 patients in this study provided written informed consent.

## 2. Information and methods

### 2.1. Study subjects

Nine patients who attended Changzhou Second People’s Hospital affiliated to Nanjing Medical University from January 2018 to January 2024, and the postoperative pathology was suggestive of intravascular smooth muscle tumor.

### 2.2. Clinical data collection

Including clinical history, gynaecological examination, imaging examination, laboratory examination, pathological results, etc. Nine patients came to the hospital for follow-up at irregular intervals after surgery, and none of them were treated with hormonal drugs during the postoperative follow-up period, and the follow-up deadline was June 6, 2024.

### 2.3. Clinical data

The median age of the 9 patients was 46 (42.5–51) years old. Three patients were admitted to the hospital because of uterine masses detected by physical examination; the remaining 6 patients were admitted to the hospital because of increased menstrual flow or abdominal pain. Nine patients did not have any obvious abnormalities of tumor indicators.

### 2.4. Treatment and follow-up

#### 2.4.1. Surgical management for pelvic-confined IVL

**Figure 1.**
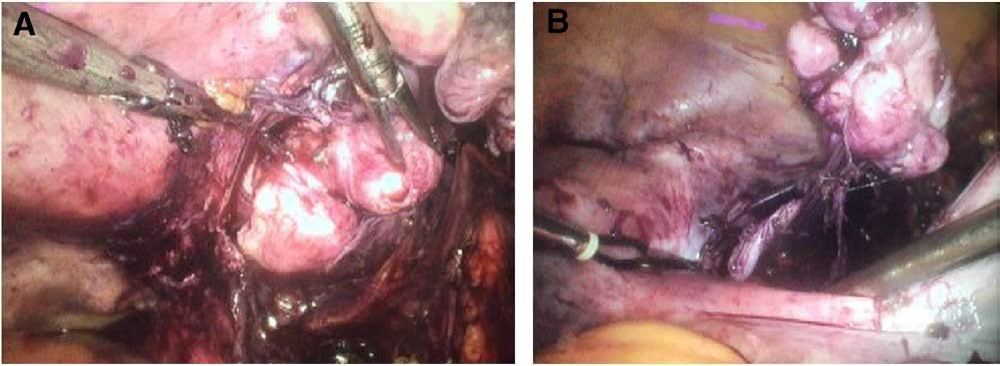
(A) The tumor extends into the right broad ligament. (B) A vascular connection between the right para-uterine tissue and tumor.

All 9 patients underwent surgical intervention. The intraoperative view is shown in Figure 1. Among them, 7 patients with lesions confined to the pelvis and no fertility desire received hysterectomy with bilateral adnexectomy (5 with bilateral salpingo-oophorectomy (BSO), 2 with bilateral adnexectomy). Intraoperative rapid pathology suggested intravascular smooth muscle tumor in 4 cases, while the remaining 3 were initially diagnosed as fibroids.

#### 2.4.2. Fertility-preserving approach

Two patients underwent laparoscopic myomectomy due to fertility desires. One of them underwent total hysterectomy 4 months postoperatively after pathological confirmation of IVL; the other declined further surgery. Both patients showed no recurrence on follow-up imaging.

#### 2.4.3. Postoperative follow-up

**Figure 2.**
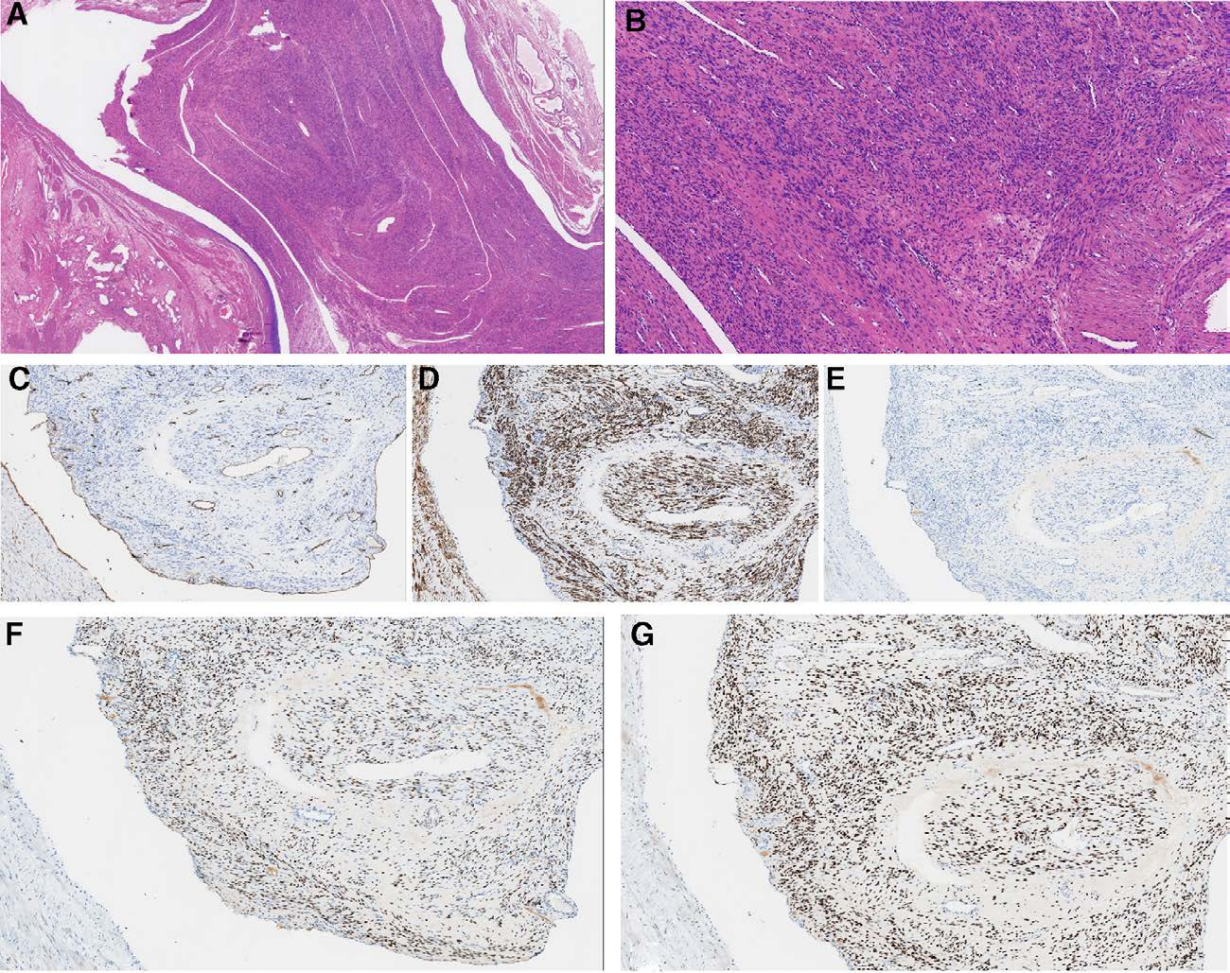
Pathological and immunohistochemical results. (A) H–E staining of tumor. Leiomyoma was seen within the vein. Magnification: X20. (B) The spindle cells have abundant cytoplasm and no obvious atypia. Magnification: X100. (C) Immunostaining by CD34 antibody confirmed that the tumor grows in the vessel. Magnification: X100. (D) Immunostaining by desmin antibody showed that desmin were diffuse positive in tumor cells. Magnification: X100. (E) Ki-67 was sporadic and isolated positive in tumor cells. Magnification: X100. (F) ER were diffuse positive in tumor cells. Magnification: X100. (G) PR were diffuse positive in tumor cells. Magnification: X100. ER = estrogen receptors, PR = progesterone receptors.

None of the patients received hormonal therapy postoperatively. During follow-up (until June 30, 2024), 8 patients who underwent hysterectomy showed no pelvic masses on ultrasound. The patient who underwent myomectomy also showed no recurrence. Two patients reported intermittent lower abdominal pain, attributed to perimenopausal pelvic inflammatory disease and managed symptomatically. The remaining 7 patients were asymptomatic (Table 1). The pathological and immunohistochemical results of 9 patients showed that leiomyoma within the vein, estrogen receptors (ER) (+), progesterone receptors (PR) (+), CD34(+), Desmin (+) and Ki-67 (±) (Fig. 2).

**Table 1:** Clinical data of 9 patients with IVL.

Case	Age	Symptom	Medical history	Operation history	Tumor size (cm)	Intraoperative view	Extrauterine extension	Operation procedure	Intraoperative rapid pathology	Surgical time (min)	Follow-up (mo)
1	46	Abnormal vaginal bleeding	Uterine leiomyoma	None	6.5	The mass extends into the right parametrial vein with a beading sign and a vascular connection between the right parametrial tissue and the mass	RBL	LM & TLH 4 months later	Fibroid tumor of the uterus	160	63 NED
2	49	Abnormal vaginal bleeding	Uterine leiomyoma	None	7.5	Dilatation of the left para-uterine vein, in which a striated solid mass was found.	None	TLH & BSO	intravascular proliferation of benign smooth muscle tissues in the left para-uterine veins.	165	59 NED
3	41	Abnormal vaginal bleeding and hypogastric painpain	None	None	Unknown	Two fibroids extending into the right broad ligament, connected on one side to the uterine vessels	RBL	Abdominal myomectomy	Degeneration of uterine fibroids	170	51 NED
4	46	Detection of a uterine mass	None	None	5.9	Interstitial leiomyoma of the anterior wall of the uterus, approximately 4.5 × 4.0 cm, with a paratesticular leiomyomatous mass attached to the uterine vessels.	None	LM& BSO	Smooth muscle tumor, multiple blood vessels seen within the tumor, fissure-like structures seen around the tumor, intraventricular smooth muscle neoplasia cannot be excluded	155	8 NED
5	51	Abnormal vaginal bleeding and hypogastric painpain	None	LM	7.5	There were more than 10 subplasma smooth muscle tumors and intermural leiomyomas of the uterus, ranging from about 1–7.5 cm in diameter, some of which were soft in texture.	None	TLH & BSO	–	110	32 NED
6	60	Postmenopausal abdominal pain	None	Unilateral salpingo-oophorectomy	6	About 8 intermural leiomyomas, subplasma smooth leiomyomas, and smooth leiomyomas of the broad ligament of the uterus, ranging in diameter from about 2.5–6.0 cm, with clear leiomyomas’ perimembranosities and a swirling structure in the cut section, and a long, striated smooth leiomyosarcoma in the ovarian vein were also seen.	RBL	TLH & LBA	Intravenous smooth muscle tumor	105	39 NED
7	42	Abnormal vaginal bleeding	None	Cesarean section	11	Multiple myometrial fibroids, grayish-white foci in the right parietal uterus, 6 × 4 × 3 cm in size, soft, with part of the tissue extending into the parietal uterine veins; varices in the left parietal uterine vessels, with very small grayish-white foci in the veins	None	TLH & BSO	Smooth muscle tumor with focal mucous degeneration	140	4 NED
8	43	Detection of a uterine mass	None	Inguinal hernia repair	5.3	Multiple earthworm-like lesions were seen on the surface of the right posterior lobe of the broad ligament on the posterior wall of the uterus, with bilateral paracervical varicose veins.	RBL	TLH & LBA	(Total uterus + bilateral fallopian tubes) Intraventricular smooth muscle neoplasia.	215	16 NED
9	51	Abnormal vaginal bleeding and hypogastric painpain	None	None	6.1	More than ten uterine myometrial and subplasma smooth muscle tumors, 1–7.5 cm in diameter, soft texture	None	TLH & BSO	–	100	45 NED

BSO = bilateral salpingo-oophorectomy, IVL = intravenous leiomyomatosis, LBA = laparoscopic bilateral adnexectomy, LM = laparoscopic myomectomy, NED = no evidence of disease, RBL = right broad ligament, TLH = total laparoscopic hysterectomy.

Our 9 patients are closely followed up after operation to date. There is no any surgical complications and no signs of recurrence. The clinical data of these patients are presented in Table 1.

## 3. Discussion

As a rare benign tumor, IVL was first discovered by Birch-Hirschfeld in 1896.^[2,3]^ As a result of the incidence of IVL is low, related researches with it are fewer, many of which are case reports or small case series dominating the literature to date. According to these reports, approximately 30% of women of reproductive age may suffer from this disease.^[4]^ Although a benign disease, its biological behavior is quasi-malignant, and can extend into the inferior vena cava, the right cardiac chambers and even the pulmonary artery, and thus induces serious complications. First intracardiac leiomyomatosis was reported in 1907.^[5]^ Currently, extrauterine involvement occurs in approximately 30% of IVL cases and intracardiac extension accounts for about 10%.^[6]^ IVL reported exclusively in women aged 28 to 80 years (median age, 45 years), and most are in the perimenopausal stage^[1]^ (see Table 2 for details). Our reports were consistent with this. The pathogenesis of IVL is unclear. Two theories are supported: one is that this tumor arises from a preexisting leiomyoma which invades venous system; the other one is that the origin of neoplasm is the wall of vessels.^[7,22]^ G. Kir et al performed immunohistochemical analysis for ER and PR at the walls of vessels in IVL, which found very low levels of ER and PR expression endothelial and subendothelial cells compared to high levels of expression of intravenous tumor cells. The results did not support the theory of vessel wall origin.^[8]^ Jing Du et al also found the identical alconclusion that supported origin arised from invasive uterine leiomyomas.^[23]^ According to our 9 patients pathology, IVL may arise from the preexisting leiomyoma in uterus. In addition, chromosomal and genetic changes may play a role in the development of IVL. Ordulu et al^[24]^ and Buza et al^[25]^ found that the gene of HMGA2 overexpressed in IVL, which might promote the growth of IVL. Besides that, the mutation of MED12 and the presence of high frequency of recurrent regional loss involving several chromosomes are also likely related to the pathogenesis of this disease^.[25]^ Mei-Jou Chen et al^[9]^ reported yet hyaluronan and its receptor, CD44, were higher levels in IVL compared to uterine leiomyoma suggesting that they may play an important role in the pathogenesis of IVL. Further studies are needed to confirm these findings.

**Table 2:** Case summary of IVL.

References	Case	Age	Symptom	Medical history	Operation history	Tumor size (cm)	Extrauterine extension	Operation procedure	Follow-up (mo)
Liu N et al^[1]^	1	34	Acute swelling in both legs and paroxysmal dyspnea for 3months	Broad ligament myoma, but pathological suspicion of IVL	TAH & LSO 18 mo ago	8	Left ovarian vein, IVC, right atrial, right ventricle	The first-stage surgery: abdominal tumor resection & RSO, The second-stage surgery: removal of the right atrial and ventricular neoplasms 2 wk later	48 NED
	2	40	Abdominal distention and bilateral leg edema	None	None	30 × 25	Pelvic venous plexus, common iliac vein, IVC, hepatic portal vein	Tumor resection + BSO	60 NED
	3	40	None, but CT revealed a tumor in the inferior vena	IVL	Myomectomy and unilateral oophorectomy 2 mo ago	10	BL, right ovarian vessels, IVC	TAH & LSO and tumor thrombectomy by angiotomy 2 mo later	2 NED
Gwacham NI et al^[2]^	1	50	Abdominal discomfort and bloat ing accompanied by lower extremity edema	A recent SARS-CoV-2 (COVID19) infection without any significant sequelae	None	20, 5.4 × 4.4	Right internal iliac vein, right common iliac vein, IVC, right atrium, right ventricle.	The single surgery: modified radical hysterectomy; right atriotomy and excision of right atrial mass; IVC venotomy and removal of the remainder of the mass	16 NED
Gunderson CC et al^[3]^	1	53	Dyspnea on exertion, 10 pound weight loss, and myalgias	Hypertension	None	6	Right the gonadal vein, IVC	TAH & BSO, sternotomy and removal of a large, mucoid inferior vena cava mass intact.	NED
Zhang L et al^[4]^	1	60	Examination revealed a right atrium neoplasm	Uterine fibroids	Hysteromyomectomy 41 yr ago	9 × 4.5 × 3	Vena iliaca interna, common iliac vein, IVC, right atrium	Unknown	None available
	2	49	Frequent chest pain after activity	Uterine fibroids	Hysteromyomectomy 2 yr ago	9 × 4.5 × 3, 4 × 3.5 × 1.5	Vena iliaca interna, vena iliaca externa, IVC, right atrium	Unknown	None available
Wei JL et al^[5]^	1	55	Dizziness	Uterine myoma, hypertension, coronary heartdisease, hyperlipidemia	TAH 7 yr ago	Unknown	Right atrium, right ventricle, 2 pulmonary arteries	Unknown	None available
Mathey MP et al^[6]^	1	52	Menometrorragia	None	None	3	None	TLH & BSO	5 NED
	2	50	Pelvic mass	None	None	7	None	TLH & BSO	12 NED
	3	38	Pelvic mass rapidly growing	None	None	10 × 8 × 8	None	TAH & BS	48 NED
	4	46	Pelvic pain	None	None	10 × 8 × 8	None	TLH & BS	LFU
	5	43	Hypermenorrhea	None	None	5	None	STLH & BS	72 NED
Kir G et al^[7]^	1	27	Menometrorragia, pelvic pain	None	None	Unknown	None	Myomectomy	12 NED
	2	21	Pelvic pain, pressure, menorrhagia	None	None	Unknown	LBL	Myomectomy, removal of br.lg.tm and GnRH agonist	6 NED
	3	46	Pelvic pain, menorragia	None	None	Unknown	None	TAH & BSO	18 NED
	4	39	Menorragia, pelvic pain	None	None	Unknown	BBL	TAH & BSO, removal of br.lg.tm	6 NED
	5	41	Pelvic pain, pressure	None	None	Unknown	None	TAH & BSO	33 NED
	6	47	Menorragia, pelvic pain	None	None	Unknown	None	TAH & BSO	24 NED
	7	42	Pelvic pain, pressure	None	None	Unknown	None	TAH & BSO	None
Du J et al^[8]^	1	54	Menorrhagia	None	None	7	None	TAH & BSO	104 NED
	2	33	Pelvic mass	None	None	8	None	Myomectomy	22, myomectomy again, then LFU
	3	37	Pelvic mass	None	None	5	BBL	TAH	76, BSO (39 after TAH)
	4	41	Pelvic mass; amenorrhea	None	None	0.5	None	TAH	77 NED
	5	47	Pelvic mass	None	None	13	LBL	TAH & LSO	73 NED
	6	47	Dysmenorrhea	None	None	3.5	None	TAH & BSO	70 NED
	7	42	Menorrhagia; pelvic mass	None	None	5	None	Subtotal hysterectomy	LFU
	8	38	Pelvic mass	None	None	1	None	TAH	58 NED
	9	52	Pelvic mass	None	None	Unknown	RBL	TAH & BSO	LFU
	10	48	Menorrhagia, pelvic mass	None	None	0.8	None	TAH	56 NED
	11	34	Pelvic mass	None	None	11	None	TAH	51, BSO (15 after TAH)
	12	41	Dysmenorrhea	None	None	2	None	TAH	34 NED
	13	47	Pelvic mass	None	None	1.5	None	TAH	34 NED
	14	40	Pelvic mass	None	None	3.5	None	TAH	33 NED
	15	42	Pelvic mass	None	None	10	LBL	TAH & BSO	29 NED
	16	50	Pelvic mass	None	None	2	RBL	TAH & BSO	27 NED
	17	50	Menorrhagia	None	None	7.5	None	TAH	27 NED
	18	46	Pelvic mass	None	None	Unknown	None	TAH	26 NED
Freitas-Ferraz AB et al^[9]^	1	52	Abrupt syncope	Unknown	Unknown	8.5 × 3.9	Right common iliac vein, IVC, right atrium, right ventricle	One-stage sugery: TAH, removal of the extrauterine and intracardiac mass	36 NED
Kong LY et al^[10]^	1	53	Irregular menstrual periods	None	None	Unknown	Right ovarian vein, IVC, right atrium	One-stage sugery: TAH & BSO, removal of inferior vena cava and right atrium mass	12 NED
Wang X et al^[11]^	1	50	Abdominal distension and shortness ofbreath for halfa year and irregular menstrual cycle	None	None	Unknown	Right ovarian vein, IVC, right atrium common iliac vein,	One-stage sugery: TAH & BSO, removal of inferior vena cava mass	6 NED
Polizzi V et al^[12]^	1	57	Chest pain, exertional dyspnea	Swollen legs, abdominal distension, syncope	None	Unknown	IVC, right atrium, right ventricle	One-stage sugery: TAH, sternotomy and removal of the inferior vena cava and intracardiac mass	None available
Schaas CM et al^[13]^	1	44	Menorrhagia	Uterine leiomyoma	Two cesarean section deliveries	10	BL	TAH & BS	NED
Grella L et al^[14]^	1	45	Syncope, palpitations, dyspnea	Uterine leiomyoma	TAH & BSO	Unknown	Hypogastric vein, IVC, right atrium, right ventricle	The first-stage surgery: sternotomy and removal of the inferior vena cava and intracardiac mass; The second-stage surgery: laparotomy and venotomy, removal of the intravenous masses 1 wk later.	7 NED
Zhang T et al^[15]^	1	41	Intermittent abdominal pain, lower limbs swell	Uterine leiomyoma	Hysterectomy	36 × 5	Left internal iliac vein, common iliac vein, right atrium	Sternotomy and laparotomy to remove the whole tumor	6 NED
Hirschowitz L et al^[16]^	1	69	Uterovaginal prolapse and fibroids	None	None	4	None	Hysterectomy	None available
	2	46	Menorrhagia and fibroids	None	None	8.5 × 3.5 × 3	None	Hysterectomy & BSO	132 NED
	3	50	Fibroids	None	None	12 × 6 × 6	None	Hysterectomy & BSO	1.5 NED
	4	41	Benign disease	None	None	2	None	Hysterectomy & BS. BO 4 mo later	8 NED
	5	45	Cystic pelvic mass	None	None	3.5	None	Hysterectomy & BSO	24 NED
Matos AP et al^[17]^	1	45	Fatigue, shortness of breath and precordial discomfort	Glaucoma, ankylosing spondylitis and multiple sclerosis	None	Unknown	pelvic veins, IVC, right cardiac chamber	Two-stage surgery	None available
Yoshida H et al^[18]^	1	34	IVL	Uterine leiomyoma	Laparoscopic myomectomy	Unknown	Right common and internal iliac veins	Removal of venous and uterine mass	22 NED
Bayramoglu D et al^[19]^	1	42	Menometrorrhagia and pelvic pain	None	None	15 × 10	Right ovarian vein, right renal vein entry of IVC	The first-stage surgery: TAH & BS & RO The second-stage surgery: removal of venous masses	12 NED
Liu HY et al^[20]^	1	50	Chest distress	None	None	17	Left ovarian vein, left renal vein, IVC, right atrium	A combined thoracic–abdominal surgery: TAH, removal of venous and intracardiac mass	None available
Clay TD et al^[21]^	1	40	Abdominal and rectal discomfort, episodes of supraventricular tachycardia requiring adenosine	Uterine leiomyoma	Myomectomy	16 × 3.5 × 2.5	Right ovarian vein, IVC, right atrium, right ventricle	A combined thoracic–abdominal surgery: TAH & BSO, removal of venous and intracardiac mass	18 NED

BBL = bilateral broad ligament, BO = bilateral oophorectomy, br.lg.tm = broad ligament tumor, BS = bilateral salpingectomy, BSO = bilateral salpingo-oophorectomy, IVC = inferior vena cava, IVL = intravenous leiomyomatosis, LBL = left broad ligament, LFU = lost to follow up, LSO = left salpingo-oophorectomy, LSO = left salpingo-oophorectomy, mo = months, NED = no evidence of disease, RBL = right broad ligament, RO = right oophorectomy, RSO = right salpingo-oophorectomy, STLH = subtotal laparoscopic hysterectomy, TAH = total abdominal hysterectomy, TLH = total laparoscopic hysterectomy.

The presentation is determined by the location and size of IVL. When it is limited in uterus, there is no obvious symptoms, and pelvic mass, uterine myoma or a broad ligament myoma is usually detected. Sometimes, patients also complain of vaginal bleeding, excessive or irregular menstruation or abdominal pain. Our 9 patients manifested abnormal menstruation or abdominal pain. Ultrasonic examination and operation both revealed uterine myoma or a broad ligament myoma. However, when this tumor extends intraluminally into the iliac vein and inferior vena cava that may induce lower extremity swelling, ascites, and Budd–Chiari syndrome owing to venous obstruction.^[2,4,6]^ When this tumor continues to progress, it can spread into the right cardiac chambers, ventricle, or even pulmonary artery, which presents abdominal distension, dizziness, shortness of breath, abrupt syncope, chest pain,congestive cardiac failure, and even sudden death.^[2,10–13]^ According to the progression of IVL, this disease can be divided into 4 stages^[14]^: stage Ⅰ: the lesion is located in uterus or parametrium, which has no obvious symptoms and is difficultly detected by imaging examination. There are a few patients complaining of pelvic pain or Irregular vaginal bleeding, etc; stage Ⅱ: the tumor extends into pelvic vessel and the affected vein may become dilated. But there is also no obvious clinical symptoms; stage Ⅲ: the tumor further spread into the inferior vena cava, renal veins or even hepatic vein, which can induce corresponding symptoms of vascular occlusion; stage Ⅳ: the tumor has reached the right atrium, and even the right ventricle, which might lead to the right ventricular dysfunction. When the tricuspid valve is completely obstructed, abrupt syncope or sudden death will occur. According to this criterion, our 9 patients were all in stage I, and they presented with irregular and excessive menstruation or abdominal pain.

Because of no obvious symptoms and extending into the small vessels of the myometrium in the early stage that is not discovered by any imaging examination, preoperative diagnosis of IVL is very difficult.^[1]^ Most patients with IVL present pelvic masses or uterine myoma, some of them extending into the broad ligament. This condition account for as high as 77%.^[1]^ So, if a pelvic mass or a broad ligament leiomyoma is found before or during surgery, we should suspect of IVL. Among our 9 patients with IVL, 2 patients were defined as broad ligament leiomyoma during pre- and intraoperation. When corresponding symptoms of vessel occlusion are present as mentioned before, IVL should be considered, and we can diagnose it by multiple imaging examinations, such as echocardiography, coronary CT, MRI. Echocardiography is mainly used to detect intracardiac leiomyomatosis, which usually shows an elongated mobile mass spreading from the inferior vena cava to the right heart chamber and can sway in synchrony with the systolic movement.^[15]^ CT or MRI is particularly used for the diagnosis of IVL as a result of high readability and large field of view. They can identify soft tissue masses within pelvic vessel, but can not distinguish tumor from thrombus. However, venography is the gold standard for identifying intravascular lesions.^[16]^ The gold standard of diagnosis of IVL is still pathology. There some researches found that certain biomarkers are positive in IVL, including desmin, PR, ER, SMA, and Ki-67, which could be used to identify this disease.^[4,26]^ According to specific pattern of growth, IVL need to be distinguished from the followed diseases^[17]^: leiomyosarcoma: it can also invade venous system. Definitive diagnosis can be made by histopathology; intravenous thrombosis: although being similar to IVL on CT, they do not enhance on contrast-enhanced MRI^[27]^; right atrial myxoma: they usually locate in cardiac chambers and do not extend into inferior vena cava. They are generally characterized by a narrow-based mass adhered to the inter-atrial septum^[28]^; and malignant tumor embolus: they mostly had a history of malignant tumor, most commonly stem from kidneys and uterus. CT and MRI are helpful for the differential diagnosis.^[27,28]^

### 3.1. Management

Currently, surgical treatment is the clinical optimal options for IVL. Complete resection of the tumor including uterus, bilateral attachments and all other lesions can achieve a favorable prognosis regarding remission and reducing the recurrence rate.^[29]^ Liang et al found that the recurrence rate of complete resection and incomplete resection were 4.29% and 37.84% respectively (*P* < .0001). Furthermore, complete resection with BSO had the lowest recurrence rate of 3.13%, incomplete resection with BSO had a progression rate of 45.45%, while incomplete resection with ovarian preservation had the highest progression rate of 75.00%.^[18]^ Thus, complete resection with BSO is essential for IVL. For the lesions being confined to the uterus and parametrium or pelvic, hysterectomy and BSO with all the other tumors through laparotomy are strongly recommended by most scholars for those patients without fertility desires.

Our 9 cases were all in stage I with lesions being confined to pelvic. Based on previous reports, 7 patients without fertility desire among them underwent hysterectomy and BSO, who were closely followed up to date after surgery with no recurrence. However, Case 3 was preoperatively diagnosed as leiomyoma, moreover, intraoperative rapid pathology also revealed leiomyoma degeneration rather than IVL. The patient and her husband requested to preserve the uterus and bilateral ovaries, so abdominal myomectomy was performed. Unfortunately, the postoperative pathological diagnosis was IVL. Therefore, a second-sugery was recommended to remove the uterus and BSO to prevent recurrence, but the patient refused. Being followed up to now, the patient with no any discomforts, which demonstrates that myomectomy may also feasible sometimes for some patients with fertility desires. This surgical approach have been performed by Yoshida H and team as well. They reported that one woman who was diagnosed with IVL after laparoscopic myomectomy through the postoperative pathology 3 years ago was admitted to their hospital for further surgery, because IVL recurred as well as had expanded into the right common and internal iliac veins. Myomectomy was performed as a result of the patient wished to preserve her fertility, and the she had a spontaneous pregnancy and successful delivery after surgery.^[30]^

However, for extra-pelvic IVL, the approach of operation includes one-stage or two-stage surgery. Two-stage surgery is that sternotomy is completed firstly, and then laparotomy at a later time. The one-stage surgery refers to laparotomy and sternotomy are both performed to removing all tumors through a single extended incision at the same time with or without cardiopulmonary bypass (CPB) and circulatory arrest. Two-stage surgery is effective and secure for those people with poor physiological conditions, lager tumors, presentation of cardiac chamber outflow obstruction and tight and extensive adhesions of tumor to the wall of intracardiac cavity and IV.^[7,19]^ Bayramoglu D et al reported a case diagnosed as extra pelvic IVL was successfully performed two-stage surgery. She was followed up for 12 mouths after operation, and complication and recurrence symptoms could not be seen.^[20]^ Whereas, two-stage surgery have some disadvantages including longer hospital stay, higher rate of recurrence, tumor embolism, and a second general anesthetic.^[7]^ With the continuous understanding of IVL, many surgeons prefered to adopt one-stage surgery which can avoid the complications and risks occurring in two-stage surgery. Deng Y and colleagues found that more patients benefited more from one-stage surgery by analyzed 110 cases defined as intravenous cardiac leiomyomatosis.^[7]^ As reported by Liu HY et al, one-stage surgery was safe and reliabale for complete resection of IVL.^[31]^ Reportedly, one-stage surgery can be achieved through a combined abdominal-thoracic or a single-abdominal incision. Li H and team devided the one-stage surgery into 4 types based on the extent of surgery, tumor extension and morphology for extra-pelvic IVL: type 1 was single laparotomy without CPB. When the maximal diameter of the proximal tumor is less than or equal to the diameter of vein at the proximal side of short venotomy, tumor can be extracted from short venotomy through single laparotomy, and then patient can avoid CPB and sternotomy; type 2 is single laparotomy with CPB. When the maximal diameter of the proximal tumor is larger than the diameter of vein at the proximal side of short venotomy, this condition usually combined adhesion between tumor and intima of IVC or heart. If tumor is pulled out blindly from the short venotomy, IVC will be teared resulting in hemodynamic instability and bleeding. To decrease the risks, CPB is indispensable; type 3 is double-incisions through mini-thoracotomy, and type 4 is through sternotomy. If the maximal diameter of tumor head is lager than the diameter of the entrance of IVC, type 4 should be opted. If not, tumor can be removed by mini-thoracotomy. Additionally, compare to the other group, blood loss, hospitalization expenses and operation time in the type 1 surgery group were significantly lesser. Hospital stay and expenses in type 2 group were significantly lesser than type 3 and type 4 groups.^[21]^ For the operation of extra-pelvic IVL, transoesophageal echocardiography is helpful, and multidisciplinary team is necessary. Because an IVL involving the IVC or heart system is closely associated with thrombosis, the anticoagulation is essential during peri-operative period.^[20]^

Although IVL has a high rate of recurrence, especially for the incomplete resection of tumor, there is no optimal mean to prevent it after operation. As ER and PR are positive, hormonal therapy using aromatase inhibitor, gonadotrophin releasing hormone agonists (GnRHa), progesterone and estrogen modulation might efficiently reduce the risk of recurrence.^[7,32]^ Low HY et al demonstrated that using GnRHa after incomplete resection of IVL was as effective as complete resection.^[29]^ However, Peng J et al found surgical type was the only factor related leading to the recurrence of IVL, and compared to hysterectomy and BSO, the recurrence risk of tumorectomy was 20 times higher, as well as GnRHa could not decrease recurrence rate.^[33]^ Doyle MP et al reported that the results of hormonal therapy were varied for residual tumor, and aromatase inhibitor is the only effective treatment to prevent the recurrence and progression of residual tumor.^[34]^ The mutation of anaplastic lymphoma kinase (ALK) gene was discovered in many tumors, such as breast caner, renal cell carcinomas, leiomyosarcoma,and non-small-cell lung cancer, which participants in the occurrence and development of tumor. Currently, Barreto-Coelho P and colleagues discovered that ALK was also overexpressed in IVL through next-generation sequencing and immunohistochemistry, and then they successfully applied the ALK targeting Crizotinib to treat disseminated IVL.^[35]^ This research suggests that precision medicine may be a new therapeutic direction for this tumor in the future.

Based on IVL hasing higher rate of recurrence, closely following up after surgery is very necessary. There is no consensus on spaced surveillance regime now. Either every 3 to 6 months or every 2 to 5 years has been recommended.^[36]^ In our 9 patients, they did not opted any adjuvant therapy after operation. But being followed up closely to date, there is no any complication or recurrence. This might be that our cases are all in an early stage and belong to stage I.

This study has several limitations. First, the sample size is small (9 cases), which may limit the generalizability of the findings. Second, all patients were in the early stage (Stage I) of IVL, and thus the management strategies discussed may not apply to advanced cases with intracardiac or pulmonary extension. Third, the follow-up period, though ongoing, remains relatively short for assessing long-term recurrence. Future multi-center studies with larger cohorts and longer follow-up are needed to validate our observations.

## 4. Conclusion

IVL is an rare variant of leiomyoma. There are 2 theories about its pathogenesis, either arising from a preexisting leiomyoma or the wall of vessel, and most scholars supported the former now. Additionally, chromosomal and genetic changes might be involved in the progression of this tumor. Preoperative diagnosis is very difficult, and pathology is the gold standard to confirm the tumor. Many researchers believed that complete resection of tumor is the optimal treatment approach. This study indicates that hysterectomy and bilateral adnexectomy could be chosen when the mass was limited to the uterus or pelvic cavity, and for patients with fertility requirements, myomectomy might also be feasible. About hormonal or the other adjuvant therapies after operation, there are still controversies, and further studies are needed to verify these findings. Closely following up is essential to timely find the operative complications and recurrence.

## Acknowledgments

We are thankful to the patients and all the physicians and technicians who participated in our study.

## Author contributions

**Conceptualization:** Junling Liu, Qiucheng Jia, Jiming Chen, Na Zhang.

**Data curation:** Xiuwen Zhang, Huimin Tang, Yuqing Cheng, Yafeng Zheng, Jiming Chen.

**Formal analysis:** Junling Liu, Hong Zheng, Yafeng Zheng.

**Funding acquisition:** Yafeng Zheng.

**Investigation:** Xiuwen Zhang, Huimin Tang.

**Software:** Huimin Tang.

**Validation:** Yuqing Cheng.

**Visualization:** Hong Zheng.

**Writing – original draft:** Junling Liu, Qiucheng Jia.

**Writing – review & editing:** Jiming Chen, Na Zhang.
